# Quantitative PCR Measurement of miR-371a-3p and miR-372-p Is Influenced by Hemolysis

**DOI:** 10.3389/fgene.2019.00463

**Published:** 2019-05-22

**Authors:** Mette Pernille Myklebust, Benedikte Rosenlund, Peder Gjengstø, Bogdan Stefan Bercea, Ása Karlsdottir, Marianne Brydøy, Olav Dahl

**Affiliations:** ^1^Department of Oncology and Medical Physics, Haukeland University Hospital, Bergen, Norway; ^2^Department of Urology, Haukeland University Hospital, Bergen, Norway; ^3^Department of Urology, Helse Fonna, Haugesund, Norway; ^4^Department of Clinical Science, University of Bergen, Bergen, Norway

**Keywords:** circulating microRNA, serum, RT-qPCR, testicular cancer, hemolysis, quality control

## Abstract

Cell-free microRNAs have been reported as biomarkers for several diseases. For testicular germ cell tumors (GCT), circulating microRNAs 371a-3p and 372-3p in serum and plasma have been proposed as biomarkers for diagnostic and disease monitoring purposes. The most widely used method for quantification of specific microRNAs in serum and plasma is reverse transcriptase real-time quantitative PCR (RT-qPCR) by the comparative Ct-method. In this method one or several reference genes or reference microRNAs are needed in order to normalize and calculate the relative microRNA levels across samples. One of the pitfalls in analysis of microRNAs from serum and plasma is the release of microRNAs from blood cells during hemolysis. This is an important issue because varying degrees of hemolysis are not uncommon in routine blood sampling. Thus, hemolysis must be taken into consideration when working with circulating microRNAs from blood. miR-93-5p, miR-30b-5p, and miR-20a-5p have been reported as reference microRNA in analysis of the miR-371a-373 cluster. We here show how these three microRNAs are influenced by hemolysis. We also propose a new reference microRNA, miR-191-5p, which is relatively stable in serum samples with mild hemolysis. In addition, we show how hemolysis can have effect on the reported microRNA levels in patient samples when these reference microRNAs are used in samples with varying levels of hemolysis.

## Introduction

MicroRNAs of the miR-371-373 cluster, which are produced in embryonal tumors, have been proposed as sensitive biomarkers for testicular germ cell tumors (GCT), both in primary cases and recurrences (Suh et al., 2004; Belge et al., 2012; Dieckmann et al., 2012; Gillis et al., 2013; Syring et al., 2015; Murray et al., 2016; Anheuser et al., 2017; Terbuch et al., 2018; Dieckmann et al., 2019). miR-371a-3p, which has the highest sensitivity and specificity of these miRNAs, has been shown to be expressed in both seminomas and non-seminomas, except pure teratomas (Dieckmann et al., 2016; Vilela-Salgueiro et al., 2018). Dieckmann et al. (2017) reported miR-371a-3p to detect GCT with sensitivity and specificity of 88.7 and 93.4%, respectively.

Detection of specific, free, circulating microRNAs in serum and plasma as biomarkers is warranted as it is a simple, non-invasive technique with little discomfort for the patient. In addition, medical staff, both in hospitals and primary health care, are trained to draw and handle blood samples. MicroRNAs have been shown to be stable in whole blood and separated serum and plasma for several hours (Mitchell et al., 2008; Glinge et al., 2017). Standard tubes and centrifugation protocols for plasma and serum collection can be used, and separated serum and plasma can be frozen for later analysis. Detection and relative quantification can be performed by real-time quantitative PCR which is well-established in molecular hospital laboratories. MicroRNAs have also been shown to have short half-life and rapid decay in circulation (Radtke et al., 2018). These features make circulating microRNA suitable as biomarkers, but still there are challenges and pitfalls. MicroRNAs are present in varying amounts in every cell of the body, including blood cells. Lysis of blood cells during phlebotomy or sample handling can therefore yield false high levels of miRNAs not reflecting the actual levels in circulation.

The minute amounts of specific microRNAs in serum and plasma need very sensitive methods for detection. Relative quantitation of microRNAs by reverse transcription real-time PCR (RT-qPCR) needs at least one reference gene in order to normalize samples, most preferably an endogenous microRNA (reference microRNA). Reference microRNAs must be expressed at similar levels throughout all samples tested, both in serum or plasma from individuals with the disease in question and in healthy controls. However, suitable reference microRNAs are often expressed at high levels in blood cells. Thus, the quantification is prone to error due to increased levels of the reference microRNA(s) caused by rupture of blood cells. Thus, hemolysis must be taken into account if the endogenous controls used are expressed in blood cells (Murray et al., 2016; Poel et al., 2018). Pre-analytical steps must be considered carefully and standardized in sampling and handling of the blood samples (Marzi et al., 2016; Pizzamiglio et al., 2017; Murray et al., 2018). There are several methods for assessment of hemolysis in serum and plasma, e.g., the ratio between miR-451 and miR-23 (Blondal et al., 2013), or absorbance at 414 nm (Kirschner et al., 2013; Shah et al., 2016).

Here we show how hemolysis affects four microRNAs used as reference genes in the quantitative analysis of miR-371a-3p and miR-372-3p. miR-93-5p, miR-20a-5p, and miR-30b-5p have been used as reference genes in analysis of the miR-371-373 cluster in serum (Gillis et al., 2013; Murray et al., 2016; Dieckmann et al., 2017). In addition, we propose a new reference microRNA, miR-191-5p. Further, we demonstrate how hemolysis will affect the calculated relative quantity (RQ) of miR-371a -3p and miR-372-3p in patient samples.

## Materials and Methods

### Blood Samples

In this study, we analyzed serum samples from two patients diagnosed with testicular germ cell cancer, Patient 1 and Patient 2. The blood was collected prior to orchiectomy in 10 mL BD Vacutainer vials with clot activator. After coagulation, serum was separated from the blood cells by centrifugation at 2000 × *g* for 10 min at 20°C within 1 h, aliquoted and frozen at –80°C. Absorbance at 414 nm was measured to assess baseline hemolysis in the serum samples. Red blood cells (RBCs) were collected from a healthy donor by centrifugation of 3 mL K_2_EDTA whole blood, with removal of plasma and buffy coat. The RBC fraction was hemolyzed by sonication. Serum was collected from the same individual and processed as described for the patient samples for use in the preparation of hemolysis standard curves described below.

No, weak and medium hemolysis in aliquots of the patient’s serum samples were imitated by adding RBC lysate to 0.0% (v/v, No hemolysis), 0.05% (Weak hemolysis) and 0.2% (Strong hemolysis) (v/v). Five replicates for each sample and hemolysis condition were performed.

### Hemolysis Serial Dilutions

Hemolysis was assessed by absorbance at 414 nm (A_414_) (Kirschner et al., 2013) in all the spiked and non-spiked serum samples using a NanoDrop 2000 spectrophotometer (NanoDrop Products). All absorbance measurements were performed in triplicates.

Standard curves for absorbance assessment of hemolyzed serum samples were made by serial dilutions of hemolyzed RBCs in serum from a healthy individual. The samples ranged from 0.0 to 1.0% (v/v) hemolysate. A_414_ was recorded prior to RNA extraction from these serum samples and followed by RT-qPCR for miR-93-5p, miR-30b-5p, miR-20a-5p, and miR-191-5p as described for the patient samples, except omission of the pre-amplification step. The experiment was repeated trice. The raw Cq-values were used to visualize how the levels of the four microRNAs increased with increasing concentration of hemolyzed RBCs in serum.

### RNA Extraction and RT-qPCR

TotalRNA including small RNAs was extracted from all samples using the miRNeasy kit (Qiagen P/N 217004, protocol RY43). A standard volume of 200 μL serum was added to 1 mL Qiazol Lysis Reagent. A mix of synthetic spike-in UniSp4 (RNA Spike-in kit, 339390, Qiagen) and 3 μg glycogen (R0551, Thermo Fisher Scientific) as carrier was added to the homogenate prior to phase-separation by chloroform. A standard volume of 500 μL upper phase was extracted and further processed as described by the manufacturer. RNA was eluted in 30 μL nuclease-free water.

Template RNA, at a fixed amount of 2 μL in each reaction, was reverse transcribed into cDNA using the Universal cDNA Synthesis kit II (Qiagen, PN 203301). In order to monitor the cDNA synthesis step, the synthetic cel-miR-39-3p (RNA Spike-in kit, Qiagen PN 339390) was included in the reaction mix of the cDNA synthesis. The final reaction mix of 10 μL was then incubated in a thermocycler at 42°C for 60 min and 95°C for 5 min. Five cDNA replicas were made for each sample and each hemolysis condition. Due to the low levels of miR-371a-372 in serum, a pre-amplification step was performed for the patient samples (Murray et al., 2011). A primer pool was assembled with the forward and reverse primers for hsa-miR-371a-3p, hsa-miR- 372-3p, miR-20a-5p, miR-30b-5p, hsa-miR-93-5p, hsa-miR-191-5p, UniSp4 and cel-miR-39-3p (all assays were miRCURY LNA miRNA PCR Assays from Qiagen, Table 1) with each assay at a final concentration of 0.1×. The reaction mix consisted of 2.5 μL 5× PerfeCTa PreAmp SuperMix (Quantabio, PN 95146), 3.125 μL primer pool, 3.1 μL cDNA (diluted 1:40) and nuclease-free water to a total volume of 12.5 μL. The pre-amplification was performed in a thermocycler with denaturation at 95°C for 10 min followed by 14 cycles of 95°C for 15 s and 60°C for 4 min. The pre-amplification product was diluted 1:5 in nuclease-free water and taken into qPCR in single assays. The qPCR reaction mix contained 5 μL 2× PerfeCTa SYBR Green Supermix (Quantabio, PN 95054), 1 μL of the respective miRCURY LNA miRNA PCR Assay (Qiagen, Table 1) and 4 μL diluted pre-amp product. The qPCR was carried out in triplicates in 384 well plates on a LightCycler480 instrument. Cycling conditions were 95°C for 10 min, 45 cycles of 95°C for 10 s and 60°C for 1 min. Melting curve analysis was performed for each well. Pipetting in qPCR was performed by robotics using a BioMek 3000 Laboratory Automation Station (Beckman Coulter).

**Table 1:** Assays used in pre-amplification and qPCR of microRNAs.

Assay	MIMAT	Cat.no, Qiagen
hsa-miR-93-5p	MIMAT0000093	YP00204715
hsa-miR-30b-5p	MIMAT0000420	YP00204765
hsa-miR-20a-5p	MIMAT0000075	YP00204292
hsa-miR-191-5p	MIMAT0000440	YP00204306
hsa-miR-371a-3p	MIMAT0000723	YP00204299
hsa-miR-372-3p	MIMAT0000724	YP00204137
cel-miR-39-3p	MIMAT0000010	YP00203952
UniSp4		YP00203953


### Quantification of MicroRNAs

The microRNAs were quantified using the comparative Ct-method (Schmittgen and Livak, 2008).

$$\Delta \Delta Cq({miR}_{i})=\left(Cq\,{sample}_{{miR}_{i}}-Cq\,{sample}_{{miR}_{ref}}\right)-\left(Cq\,{cal}_{{miR}_{i}}-Cq\,{cal}_{{miR}_{ref}}\right)$$

$$Where\;{miR}_{i}=\text{microRNA of interest, }{miR}_{ref}=\text{reference miR, sample=patient sample and cal=calibrator sample.}$$

The calibrator sample was a patient sample with very low levels of miR-371a-3p and miR-372-3p. RQs are 2^-ΔΔCq^.

### Statistical Tests and Ethics

The study was approved by the Regional Ethics Committee of Central Norway (REK Midt, 2015/1475). Patients included in the study gave written informed consent in accordance with the Declaration of Helsinki. All statistical tests were performed in IBM SPSS Statistics Version 25. Expression levels of the microRNAs were compared using the Student’s *t*-test with Levene’s *F*-test for equality of variances. *P*-values < 0.05 in the two tailed *t*-tests were considered significant.

## Results

### Hemolysis and Reference MicroRNAs’ Levels

**FIGURE 1:**
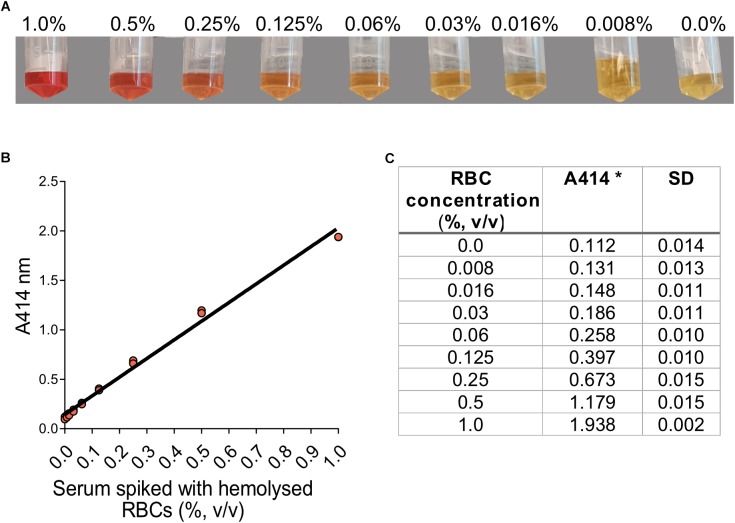
Visual inspection and absorbance measurement of hemolyzed red blood cells in serum. **(A)** Serum with low initial absorbance at 414 nm was spiked with varying amounts of hemolyzed RBCs. The percentage indicated is v/v. **(B)** Absorbance at 414 nm for the different concentrations of hemolyzed RBCs in serum. The three replicates for each concentration are plotted. **(C)** A414 at different RBC-concentrations (%, v/v) in serum.

The serial dilution of hemolyzed RBCs in serum showed that the amounts of RBCs added ranged from a non-detectable to a strong red color by visual inspection (Figure 1A). By eye, hemolysis could be detected at approximately 0.06% (v/v) RBCs in blood, but the hemolysis did not appear pronounced until 0.25% (v/v). The absorbance measurement at 414 nm showed a linear absorbance increase in the analyzed range (0–1.0% v/v) RBCs in serum (Figure 1B,C).

**FIGURE 2:**
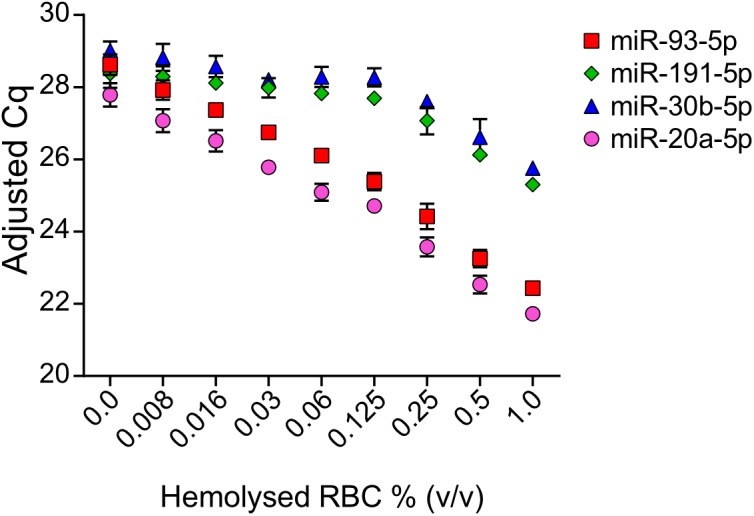
Raw Cq-values of endogenous miR-191-5p, miR-93-5p, miR-30b-5p, and miR20a-5p in serum spiked with hemolyzed RBCs. Values shown are mean Cq-values with SD from the three replicates. Raw Cq-values were adjusted according to UniSp4 in order to correct for variations in RNA extraction efficiency.

Quantification by RT-qPCR of the miRNAs proposed as endogenous controls in analysis of microRNAs 371a-372 in serum showed that the concentration of miR-191-5p and miR-30b-5p did increase in serum when hemolysis occurs, but the increase in miR-93-5p and miR-20a-5p were more pronounced (Figure 2). According to the Cq-values shown in Figure 2, the levels of miR-93-5p and miR-20a-5p increased in serum from the lowest amount of 0.008% added RBCs. The increase in miR-93-5p and miR-20a-5p appeared almost linear. There was a small increase in miR-191-5p and miR-30b-5p in serum when 0.008% hemolyzed RBCs were added, but the raw Cqs were quite stable until 0.125% RBCs were added. Hence, according to our results miR-191-5p and miR-30b-5p were the microRNAs that were the least influenced by hemolysis among the four microRNAs tested. miR-93-5p and miR-20a-5, on the other hand, are abundant in blood cells and even very weak hemolysis cause a measurable increase of these microRNAs in serum.

### Hemolysis and miR-371a- 372 Levels in Patient Samples

Raw Cq-values from RT-qPCR analysis of miR-30b-5p, miR-93-5p, miR-191-5p, miR-371a-3p, and miR-372-3p adjusted for variation in RNA extraction efficiencies according to UniSp4 are shown in Supplementary Figure S1. Some variation was seen in the RNA extraction efficiency (UniSp4, CV 3.99%), while the cDNA synthesis step was stable (cel-miR-39-3p, CV 1.69%) across samples.

**FIGURE 3:**
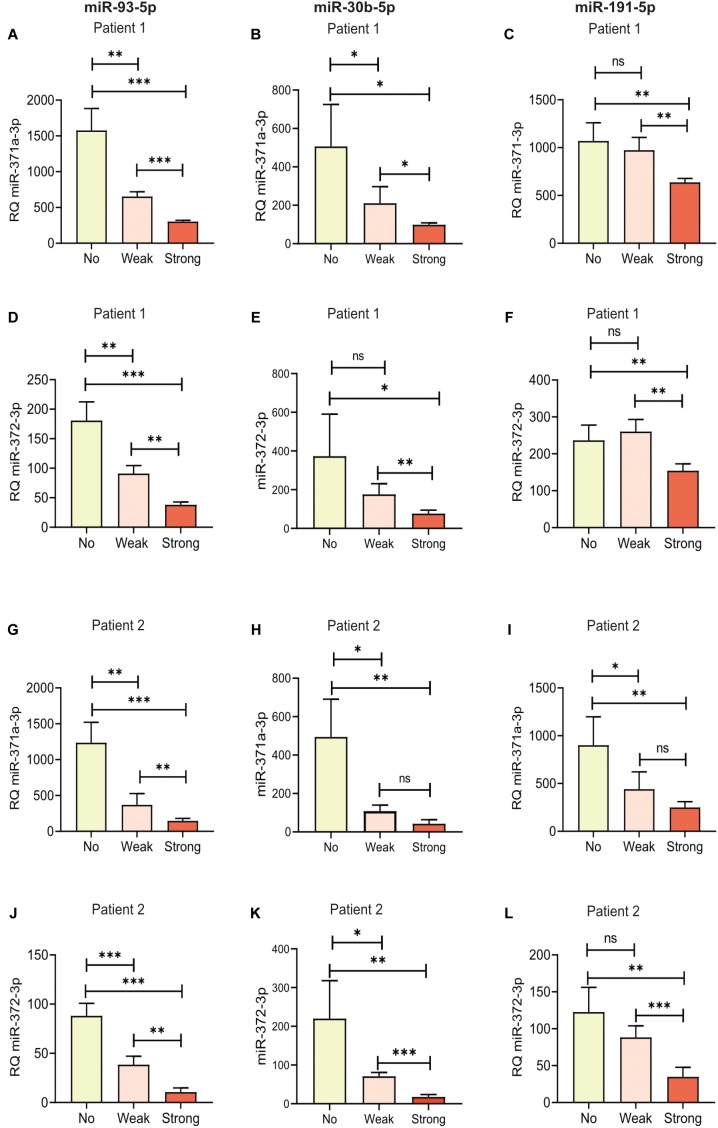
Hemolysis affected the measured levels of specific microRNAs in serum from the two patients, Patient 1 and Patient 2. Relative quantities (RQs) representing miR-371a-3p and miR-372-3p were calculated with miR-93-5p **(A,D,G,J)**, miR-30b-5p **(B,E,H,K)** and miR-191-5p **(C,F,I,L)** as reference genes. “No,” “Weak,” and “Strong” represent the same serum, but with 0.0, 0.05, or 0.2% added RBCs, respectively. ^∗^*P* < 0.05, ^∗∗^*P* < 0.01, ^∗∗∗^*P* < 0.001. Bars represent mean RQ with standard deviations (SD). RQ = 2^-ΔΔCq^.

As described, two different patient samples were spiked with increasing amounts of hemolyzed RBCs. We measured the initial absorbance at 414 nm for the patient samples and again after spiking (Table 2). The RBC fraction used to spike samples did not contain any miR-371a-3p or miR-372-3p (Cq > 40, results not shown). miR-371a-372 levels were higher in Patient 1 than in Patient 2 (Figure 3). All RQs with 95% confidence intervals (95% CI) are available in Supplementary Table S1. The miR-371a-3p and miR-372-3p RQs for Patient 1 and Patient 2 are statistically significant lower in the spiked samples than in the sample with no added RBCs. As shown in Figure 3A,D,G,J, the RQs of both miR-371a-3p and miR-372-3p were statistically significant lower in the samples with added RBCs than in the sample with no added RBCs when miR-93-5p was used as endogenous control (*P* < 0.05). The differences in RQs between the “Weak” and “Strong”-hemolysis samples are also statistically significant lower (*P* < 0.05). When miR-30b-5p was used as endogenous control in these samples, the differences in RQs in “No hemolysis” versus “Weak hemolysis” were lower, but still statistically significant for miR-371a-3p in both Patient 1 and Patient 2 (Figure 3B,H, *P* = 0.036 and *P* = 0.011, respectively) and for miR-372-3p in Patient 2 (Figure 3E, *P* = 0.010), but not significant for Patient 1 (Figure 3K, *P* = 0.112). miR-191-5p as reference microRNA gave miR-371a-5p and miR-372-5p RQs that were not statistically significant higher in the “No hemolysis” versus the “Weak hemolysis” samples (*P* > 0.05, Figure 3C,F,L), except for miR-371a-3p in Patient 2 (Figure 3I, *P* = 0.023).

**Table 2:** Absorbance at 414 nm in patients’ serum before and after spiking with RBCs.

	No added RBCs	0.05% RBC added:	0.2% RBCs added
Sample	(no hemolysis)	(weak hemolysis)	(strong hemolysis)
Patient 1	0.148	0.222	0.330
Patient 2	0.120	0.154	0.292


**FIGURE 4:**
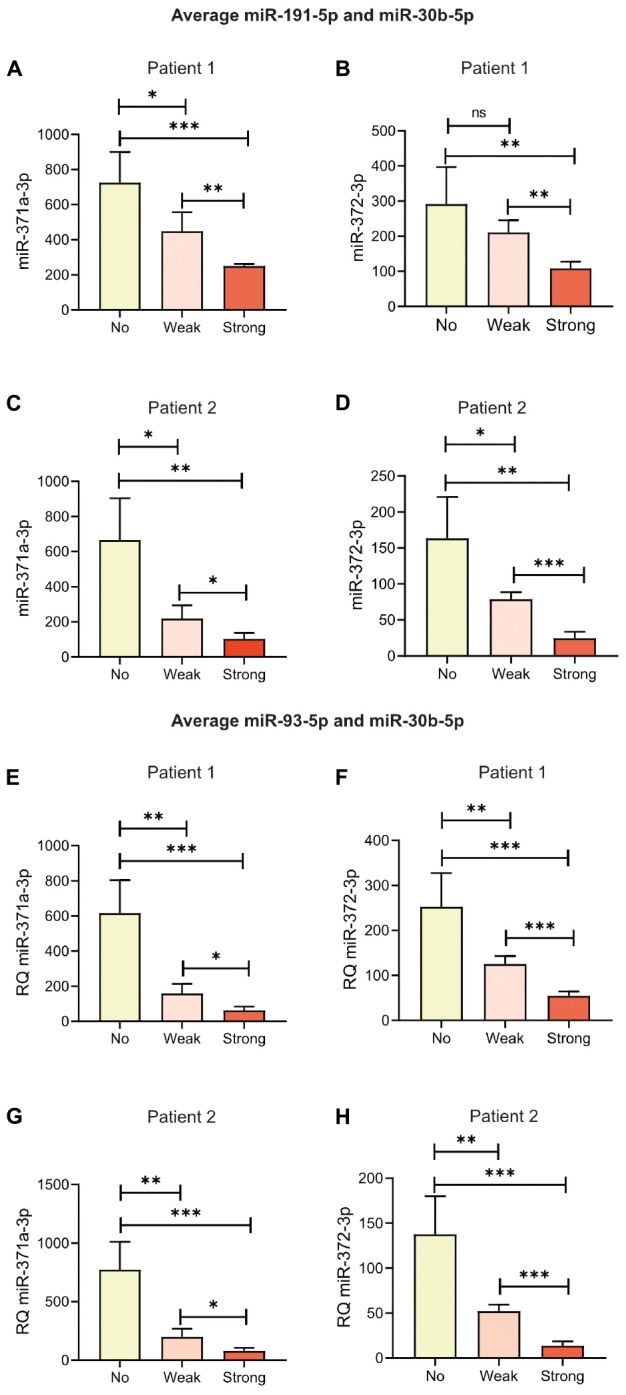
Hemolysis affected the measured levels of specific microRNAs in serum from the two patients. Relative quantities (RQs) representing miR-371a-3p and miR-372-3p in Patient 1 and Patient 2 were calculated with the average of miR-191-5p and miR-30b-5p **(A–D)** or the average of miR-30b-5p and miR-93-5p **(E–H)** as reference genes. “No,” “Weak,” and “Strong” represent the same serum, but with 0.0, 0.05, or 0.2% added RBCs, respectively. ^∗^*P* < 0.05, ^∗∗^*P* < 0.01, ^∗∗∗^*P* < 0.001. Bars represent mean RQ with standard deviations (SD). RQ = 2^-ΔΔCq^.

Regarding the differences in RQ for both miR-371a-3p and miR-372-3p between “No hemolysis” and “Strong hemolysis,” they were statistically significant in all samples for all the tested reference microRNAs (Figure 3A–L, *P* < 0.05). It is generally accepted that the mean of at least two reference genes should be used in relative quantification of microRNAs. We therefore calculated the RQs for miR-371a-3p and miR-372-3p with the average of the two best reference microRNAs, miR-30b-5p and miR-191-5p (Figure 4A–D). As expected, the results were in line with the results for miR-30b-5p and miR-191-5p used as reference genes alone. The differences in RQs between “No hemolysis” and “Weak hemolysis” were smaller than when miR-30b-5p and miR-93-5p were used in the analysis of miR-371a-372 (Figure 4E–H).

## Discussion

miR-371a-3p, miR-372-3p, and miR-373-3p are promising biomarkers for detection and surveillance in testicular germ cell cancer. miR-371a-3p in particular has been shown to have high sensitivity and specificity (Gillis et al., 2013; Anheuser et al., 2017; Dieckmann et al., 2017; van Agthoven et al., 2017; Radtke et al., 2018). We have here shown how hemolysis can have impact on quantification of microRNAs in serum and the possible influence on clinical decisions if not interpreted correctly.

Circulating miRNAs are interesting as biomarkers because the sampling is easily standardized and no invasive interventions are needed. The most used laboratory technique for targeted detection and quantification of low numbers of microRNAs is relative quantification by RT-qPCR (Schmittgen and Livak, 2008). Consensus is that in order to achieve robust and reproducible relative quantification of mRNAs and microRNAs, normalization against at least two endogenous reference miRNAs expressed at stable levels across the samples in question should be used to correct for variations in sample composition, non-endogenous variation expression introduced during RNA extraction, RNA handling and storage and the RT-qPCR protocol (Bustin et al., 2009). The challenge in analysis of circulating microRNAs is to identify such stably expressed endogenous reference genes. Most of the microRNAs present at relatively high levels in serum and plasma are also present in blood cells and will be released to serum and plasma during cell lysis. When working with serum and plasma from blood samples, contamination with RBCs, WBCs or platelets can therefore introduce variation and possibly false conclusions. Weak hemolysis of RBC *in vitro* during blood sampling are relatively common. We have in this work shown how hemolysis even in the sub-visible range can lead to large differences in measured miR-371a-3p and miR-372-5p levels, even though the levels of these two microRNAs themselves are not influenced by hemolysis.

There is an ongoing debate on how to normalize the expression of the microRNAs in question during calculation of RQ using the ΔΔCt-method. Three main strategies have been proposed, i.e., normalization to geometric mean (Vandesompele et al., 2002), normalization to exogenic reference miRNA e.g., cel-miR-39-5p (Xu et al., 2014) and normalization to stably expressed internal reference microRNAs (Bustin et al., 2009). Normalization to the geometric mean applies to studies analyzing high numbers of miRNA targets e.g., miRNA sequencing or arrays and is not suitable to studies where only a few miRNAs are analyzed. Levels of exogenic reference miRNAs added to serum prior to RNA extraction will not be changed by hemolysis, but this method is prone to pipetting variation and batch variations and will not correct for biological variation in overall microRNA levels in the samples. Quantification of microRNA using the standard curve method in RT-qPCR is not commonly used due to the challenges in concentration measurement of the small RNAs. We find in our study that miR-191-5p alone is the best reference microRNA. Our study is focused on hemolysis and miR-191-5p levels in serum may be influenced by other physiological conditions not studied by us. In addition, this microRNA is highly abundant in serum and might not be the best reference microRNA when studying microRNAs expressed at very low levels in serum.

Other groups have also argued that hemolysis must be monitored when working with quantitative microRNA analysis in serum or plasma (Blondal et al., 2013; Murray et al., 2016). Different assays for hemolysis assessment are used, e.g., the miR-451a-miR-23a-3p ratio (Blondal et al., 2013), hemoglobin concentration measurement (Shah et al., 2016), absorbance at 414 nm (Kirschner et al., 2013). We chose to use absorbance measurement at 414 nm (A414) in our study because the assessment is very cost effective, requires minimal amounts of serum and can be performed without further sample processing, e.g., sample quality can be assessed prior to RNA extraction. We also based our choice of A414 as the preferred method for hemolysis detection on the finding of Shah et al where A414 detects hemolysis down to 0.004% and correlates well with the miR-451a/23a ratio (Shah et al., 2016). Our results clearly show that hemolysis must be taken into consideration when working with microRNAs in serum and plasma. Contamination of the sample with blood cells containing any of the microRNAs analyzed can interfere with the results, even if the hemolysis is mild and the microRNA that increase due to hemolysis is not the main microRNA in question, but used as a reference microRNA in calculation of RQ. Of note, hemoglobin is also a known inhibitor in PCR, but the concentrations of hemoglobin must be higher than in our study to cause inhibition (Al-Soud and Radstrom, 2001; Sidstedt et al., 2018). In our case where the microRNAs increased by hemolysis were not the main microRNA analyzed, but used as reference microRNA in normalization during relative quantification by RT-qPCR, the result will be a falsely low RQ calculated for the miRNA in question, here miR-371a-3p and miR-372-3p. Hypothetically as a worst case scenario, a patient sample expressing low, but clearly true positive levels of miR-371a-3p, could by barely visible hemolysis be concluded to have miR-371a-3p within the normal reference range and thus reported as non-pathological. Another hypothetical case is that during monitoring disease activity, the conclusion based on a hemolyzed sample could be decline in miR-371a-3p after treatment and thus effect of treatment. The conclusion based on the true, unchanged miR-371a-3p value would have been no effect of treatment.

Analysis of cell-free microRNAs can be performed by other quantitative techniques than RT-qPCR, e.g., Next Generation Sequencing (NGS) and digital droplet PCR (ddPCR). Especially ddPCR holds great promise in analysis of rare targets with better precision and reproducibility, but equivalent sensitivity compared to RT-qPCR (Hindson et al., 2013). DdPCR is an endpoint analysis with partition through dilution and is thus less prone to inhibitors compared to RT-qPCR (Dingle et al., 2013). Another advantage of ddPCR is that the technique provides absolute quantification without a calibration curve or calibrator sample (as used in RQ). Some also argue that the endogenous control(s) can be omitted in microRNA quantification using ddPCR or NGS, but we argue that appropriate endogenous controls must be used in order to correct for non-biological variation among study samples e.g., differences in RNA extraction efficiencies and possible degradation of samples during storage, as is also proposed by others (Stein et al., 2017). Using ddPCR for analysis of miR-371-373 by NGS or ddPCR may improve the technical performance of the microRNA detection, but hemolysis must still be accounted for as long as microRNAs present in the RBCs are used as endogenous controls.

To conclude, hemolysis assessment is an important quality control when working with circulating microRNAs in serum and plasma. Visual inspection is not sufficiently sensitive for monitoring hemolysis. Further efforts for finding good reference genes and normalization strategies in RT-qPCR analysis of circulating microRNAs must go on until consensus is reached if these microRNAs are to be used quantitatively for diagnostic purposes and clinical decisions.

## Data Availability

The raw data used for calculations are available in Supplementary Data File S1.

## Ethics Statement

This study was carried out in accordance with the recommendations of Helse Vest HF’s guidelines and the Regional Ethics Committee of Central Norway with written informed consent from all subjects. All subjects gave written informed consent in accordance with the Declaration of Helsinki. The protocol was approved by the Regional Ethics Committee of Central Norway.

## Author Contributions

MM, BR, and OD contributed in conception, design, interpretation of results and drafting of the manuscript. MM and BR performed the laboratory work and statistical analyses. PG, BB, ÁK, and MB included patients. All authors contributed in manuscript revision.

## Conflict of Interest Statement

The authors declare that the research was conducted in the absence of any commercial or financial relationships that could be construed as a potential conflict of interest.

## Supplementary Material

Figure S1 Cq-values for UniSp4, cel-miR-39-3p, miR-93-5p, miR-30b-5p, miR-371a-3p and miR-372-3p in two patient samples spiked with hemolyzed RBCs to resemble no (0%), weak (0.05%) or strong (0.2%) hemolysis. Values shown are mean with range from five replicates for each sample and hemolysis condition.

Table S1 Mean Cq-values with SD for samples and hemolysis conditions presented in Figure 3, 4.

DATA FILE S1 Raw data used for calculations in this study.
