# Supplementary figures and images for: Quantitative PCR Measurement of miR-371a-3p and miR-372-p Is Influenced by Hemolysis
Source: Front Genet. 2019 May 22;10:463. doi: 10.3389/fgene.2019.00463 (PMC6539204; doi:10.3389/fgene.2019.00463)

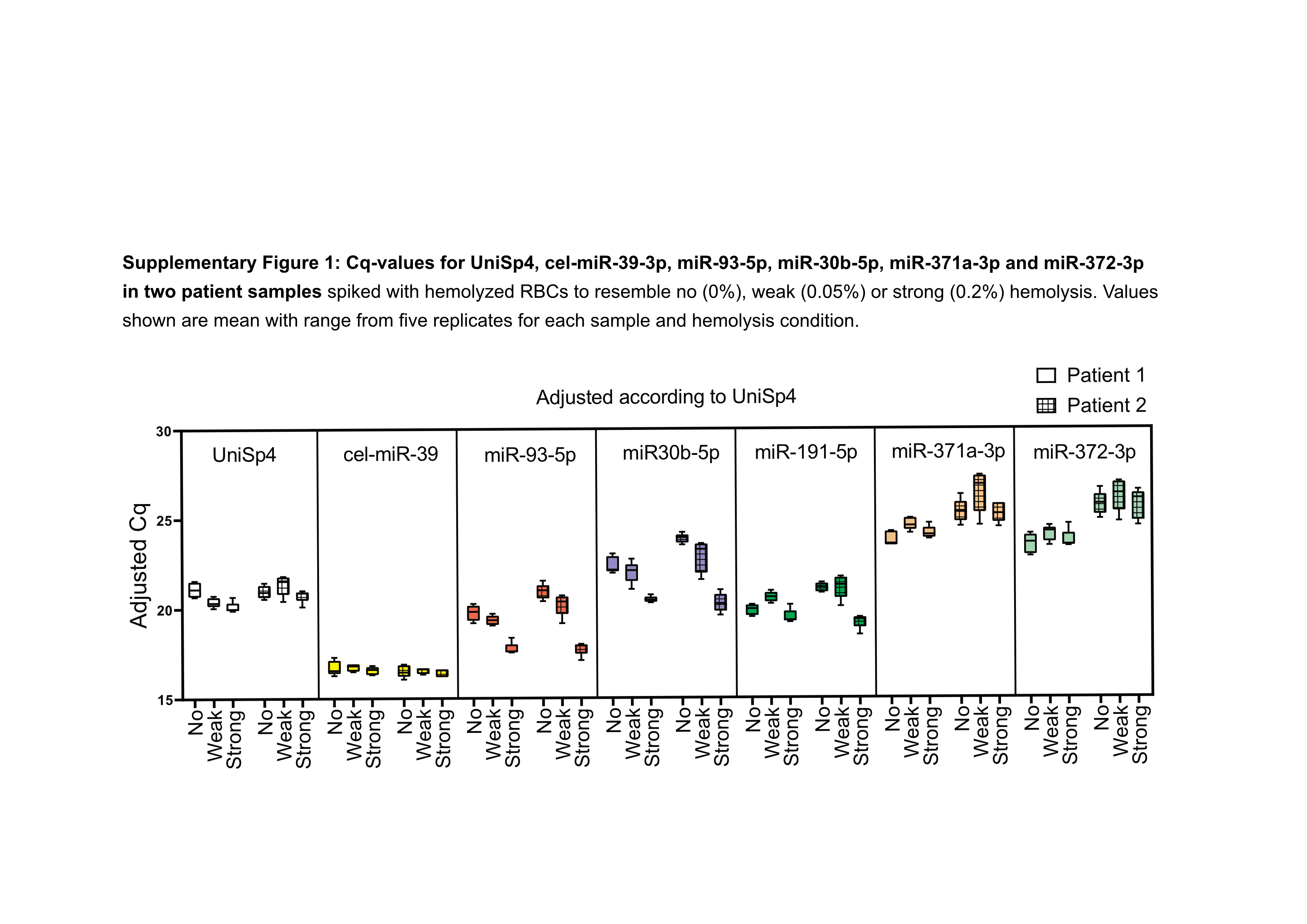

Supplement: Figure S1 — Cq-values for UniSp4, cel-miR-39-3p, miR-93-5p, miR-30b-5p, miR-371a-3p and miR-372-3p in two patient samples spiked with hemolyzed RBCs to resemble no (0%), weak (0.05%) or strong (0.2%) hemolysis. Values shown are mean with range from five replicates for each sample and hemolysis condition. [file Image_1.tiff]
