# Supplementary material for: Quantitative PCR Measurement of miR-371a-3p and miR-372-p Is Influenced by Hemolysis
Source: Front Genet. 2019 May 22;10:463. doi: 10.3389/fgene.2019.00463 (PMC6539204; doi:10.3389/fgene.2019.00463)
Supplement: Table S1 — Mean Cq-values with SD for samples and hemolysis conditions presented in Figure 3, 4. [file Table_1.docx]

**Supplementary table 1-1**: RQ-values of Patient 1 and Patient 2 for miR-371a-3p and miR-372-3p across no, weak and strong hemolysis. MiR-93-5p alone used as reference microRNA.

| **Sample** | **miR-371a-3p**  **Mean RQ (95% CI)** | **miR-372-3p**  **Mean RQ (95% CI)** |
| --- | --- | --- |
| Patient 1 No hemolysis^1^ | 1576 (1195-1958) | 181 (142-220) |
| Patient 1 Weak hemolysis^2^ | 653 (570– 735) | 91 (74-108) |
| Patient 1 Strong hemolysis^3^ | 302 (279-326) | 38 (32-44) |
| Patient 2 No hemolysis | 1267 (884-1589) | 88 (72-104) |
| Patient 2 Weak hemolysis | 368 (171-565) | 38 (28-49) |
| Patient 2 Strong hemolysis | 145 (100-190) | 11 (5-16) |

^1^No hemolysis: No RBCs added.
^2^Weak hemolysis: 0.05%(v/v) hemolyzed RBCs added.
^3^Strong hemolysis: 0.2% (v/v) hemolyzed RBCs added.

**Supplementary table 1-2**: RQ-values of Patient 1 and Patient 2 for miR-371a-3p and miR-372-3p across no, weak and strong hemolysis. MiR-30b-5p alone used as reference microRNA.

| **Sample** | **miR-371a-3p**  **Mean RQ (95% CI)** | **miR-372-3p**  **Mean RQ (95% CI)** |
| --- | --- | --- |
| Patient 1 No hemolysis^1^ | 506 (234-778) | 373 (103-643) |
| Patient 1 Weak hemolysis^2^ | 210 (102-318) | 175 (106-244) |
| Patient 1 Strong hemolysis^3^ | 98 (85-111) | 77 (55-98) |
| Patient 2 No hemolysis | 494 (250-738) | 220 (98-341) |
| Patient 2 Weak hemolysis | 108 (69-148) | 71 (58-83) |
| Patient 2 Strong hemolysis | 43 (16-69) | 18 (10-25) |

^1^No hemolysis: No RBCs added.
^2^Weak hemolysis: 0.05%(v/v) hemolyzed RBCs added.
^3^Strong hemolysis: 0.2% (v/v) hemolyzed RBCs added.

**Supplementary table 1-3**: RQ-values of Patient 1 and Patient 2 for miR-371a-3p and miR-372-3p across no, weak and strong hemolysis. MiR-191-5p alone used as reference microRNA.

| **Sample** | **miR-371a-3p**  **Mean RQ (95% CI)** | **miR-372-3p**  **Mean RQ (95% CI)** |
| --- | --- | --- |
| Patient 1 No hemolysis^1^ | 1069 (833-1305) | 236 (185-288) |
| Patient 1 Weak hemolysis^2^ | 974 (808-1140) | 260 (219-301) |
| Patient 1 Strong hemolysis^3^ | 637 (586-688) | 154 (131-177) |
| Patient 2 No hemolysis | 900 (530-1271) | 123 (81-164) |
| Patient 2 Weak hemolysis | 440 (215-666) | 88 (69-108) |
| Patient 2 Strong hemolysis | 249 (174-325) | 35 (19-51) |

^1^No hemolysis: No RBCs added.
^2^Weak hemolysis: 0.05%(v/v) hemolyzed RBCs added.
^3^Strong hemolysis: 0.2% (v/v) hemolyzed RBCs added.

**Supplementary table 1-4**: RQ-values of Patient 1 and Patient 2 for miR-371a-3p and miR-372-3p across no, weak and strong hemolysis. Average of miR-191-5p and miR-30b-5p used as reference microRNA.

| **Sample** | **miR-371a-3p**  **Mean RQ (95% CI)** | **miR-372-3p**  **Mean RQ (95% CI)** |
| --- | --- | --- |
| Patient 1 No hemolysis^1^ | 725 (508-942) | 292 (161-422) |
| Patient 1 Weak hemolysis^2^ | 448 (313-584) | 211 (168-253) |
| Patient 1 Strong hemolysis^3^ | 250 (234-266) | 108 (85-132) |
| Patient 2 No hemolysis | 665 (369-961) | 163 (92-235) |
| Patient 2 Weak hemolysis | 218 (124-312) | 79 (67-91) |
| Patient 2 Strong hemolysis | 103 (59-147) | 25 (14-36) |

^1^No hemolysis: No RBCs added.
^2^Weak hemolysis: 0.05%(v/v) hemolyzed RBCs added.
^3^Strong hemolysis: 0.2% (v/v) hemolyzed RBCs added.

**Supplementary table 1-5**: RQ-values of Patient 1 and Patient 2 for miR-371a-3p and miR-372-3p across no, weak and strong hemolysis. Average of miR-93-5p and miR-30b-5p used as reference microRNA.

| **Sample** | **miR-371a-3p**  **Mean RQ (95% CI)** | **miR-372-3p**  **Mean RQ (95% CI)** |
| --- | --- | --- |
| Patient 1 No hemolysis^1^ | 867 (672-1062) | 252 (159-346) |
| Patient 1 Weak hemolysis^2^ | 365 (270-459) | 125 (103-147) |
| Patient 1 Strong hemolysis^3^ | 172 (157-186) | 54 (42-66) |
| Patient 2 No hemolysis | 773 (479-1068) | 138 (85-190) |
| Patient 2 Weak hemolysis | 198 (112-285) | 52 (43-61) |
| Patient 2 Strong hemolysis | 78 (44-112) | 14 (8-20) |

^1^No hemolysis: No RBCs added.
^2^Weak hemolysis: 0.05%(v/v) hemolyzed RBCs added.
^3^Strong hemolysis: 0.2% (v/v) hemolyzed RBCs added.
